# Doping-Free Arsenene Heterostructure Metal-Oxide-Semiconductor Field Effect Transistors Enabled by Thickness Modulated Semiconductor to Metal Transition in Arsenene

**DOI:** 10.1038/s41598-019-40675-7

**Published:** 2019-03-08

**Authors:** Dongwook Seo, Jiwon Chang

**Affiliations:** Department of Electrical and Computer Engineering, Ulsan National Institute of Science and Technology (UNIST), Ulsan, 44919 South Korea

## Abstract

Two-dimensional (2-D) materials such as MoS_2_ and phosphorene provide an ideal platform to realize extremely thin body metal-oxide-semiconductor field effect transistors (MOSFETs) which is highly immune to short channel effects in the ultra-scaled regime. Even with the excellent electrostatic integrity inherent in 2-D system, however, 2-D materials suffer from the lack of efficient doping method which is crucial in MOSFETs technology. Recently, an unusual phase transition from semiconductor to metal driven by the thickness modulation has been predicted in mono-elemental 2-D material arsenene. Utilizing this extraordinary property, we propose doping-free arsenene heterostructure MOSFETs based on the lateral multilayer (metallic source)/monolayer (semiconducting channel)/multilayer (metallic drain) arsenene heterostructure. Metallic multilayer arsenene in the source and drain can serve as electrodes without doping. We investigate the potential performance of arsenene heterostructure MOSFETs through atomistic simulations using density functional theory and nonequilibrium Green’s function. The intrinsic upper limit of the on-state current in arsenene heterostructure MOSFETs is estimated by studying the effect of layer number in the source and drain. We comprehensively analyze the competitiveness of arsenene heterostructure MOSFETs through benchmarking with monolayer arsenene homostructure MOSFETs equipped with the highly degenerate doped source and drain, suggesting superior performance of heterostructure MOSFETs over homostructure MOSFETs.

## Introduction

Over the past few years, gapped two-dimensional (2-D) materials represented by MoS_2_^1,2^ and phosphorene^3,4^ have been widely explored for the future device applications. Such 2-D materials are attractive candidates especially for the channel materials in the aggressively scaled metal-oxide-semiconductor field effect transistors (MOSFETs) since their extreme thinness allows almost ideal electrostatic control over the channel, making them robust to short channel effects^1–7^. However, one of the critical issues hindering 2-D materials from the adoption in the MOSFETs technology is the challenge to dope 2-D materials effectively to n- or p-type, which lies in the heart of MOSFETs technology. In the conventional silicon MOSFETs, highly doped source and drain electrodes are achieved by the substitutional impurity atoms. However, applying such a technique to 2-D material MOSFETs is challenging because of the atomically thin nature of 2-D materials. In search for an alternative doping strategy, charge transfer doping using deposition of molecules or adatoms on 2-D materials has been suggested by theoretical calculations^8,9^ and experimentally demonstrated^10–12^. Such doping methods using surface modification by molecules or adatoms can locally alter the conductivity of 2-D materials but the long-term stability in the ambient atmosphere still remains as an issue.

Recently, new mono-elemental 2-D materials, namely arsenene and antimonene made of As and Sb, respectively, have emerged as promising materials^13–17^. It is theoretically predicted that monolayer of arsenene and antimonene has a sizable band gap^13–17^ and comparable or superior mobility than MoS_2_ and phosphorene^17^. *Ab-initio* quantum transport simulations reveal that monolayer of arsenene and antimonene are competitive channel materials for the deeply scaled MOSFETs^17,18^. In experiments, a few layers of arsenene and antimonene have been successfully synthesized on various substrates^19–22^. Moreover, even with the concern about the oxidation of antimonene under the ambient condition predicted by the *ab-initio* calculation^23^, high stability upon the exposure to air and water has been experimentally proven^20–22,24^. Other than the excellent mobility and stability, the most unique property of arsenene and antimonene is the abrupt switching of electronic properties from semiconducting to metallic depending on the thickness of layers. Monolayer of arsenene and antimonene has a band gap larger than 1 eV while the band gap is entirely closed in multilayer due to the strong interaction between different layers^13,16^. This thickness modulated phase transition which has never been observed in the other materials before can provide revolutionary solutions to the critical challenges such as the low on-state current issue in tunneling field-effect transistors (TFETs) as discussed in our previous work^25^. In the present work, we focus on the possibility to utilize this property to resolve the doping issue in 2-D materials MOSFETs. We can consider using metallic multilayer for the source and drain electrodes and semiconducting monolayer for the channel in MOSFETs. Then, this heterostructure MOSFETs will not require any doping processes to form electrodes since multilayer itself is already highly conductive. Similar concept of device has been demonstrated in the other 2-D material MOSFETs based on MoS_2_ which also exhibits the phase transition triggered by a crystal structure change from trigonal prismatic (2 H) to octahedra (1 T)^26^. ref.^26^ reports substantial performance improvement in MoS_2_ MOSFETs using metallic 1 T phase MoS_2_ for the source and drain regions. However, the stability of 1 T phase electrodes under the high-performance device operation is still unknown since 1 T phase MoS_2_ is metastable^26^ and forming local 1 T phase MoS_2_ in the source and drain needs an additional chemical process. For arsenene and antimonene, the metallic nature of multilayer is energetically stable since it originates from the interlayer coupling and the multilayer/monolayer heterostructure can be obtained by selective etching, a well-known approach to control the nanostructure thickness. Atomic layer etching^27–29^, recently demonstrated for other 2-D materials such as graphene^27^ and MoS_2_^28,29^, may be applicable to arsenene and antimonene to control the layer number accurately.

Herein, potential performance of doping-free arsenene heterostructure MOSFETs is investigated via density functional theory (DFT) and nonequilibrium Green’s function (NEGF) calculations. We focus on only arsenene since band structures of arsenene and antimonene are quite alike and hence almost the same level of device performances is expected as discussed in monolayer arsenene and antimonene MOSFETs^17^.

## Results and Discussion

**Figure 1:**
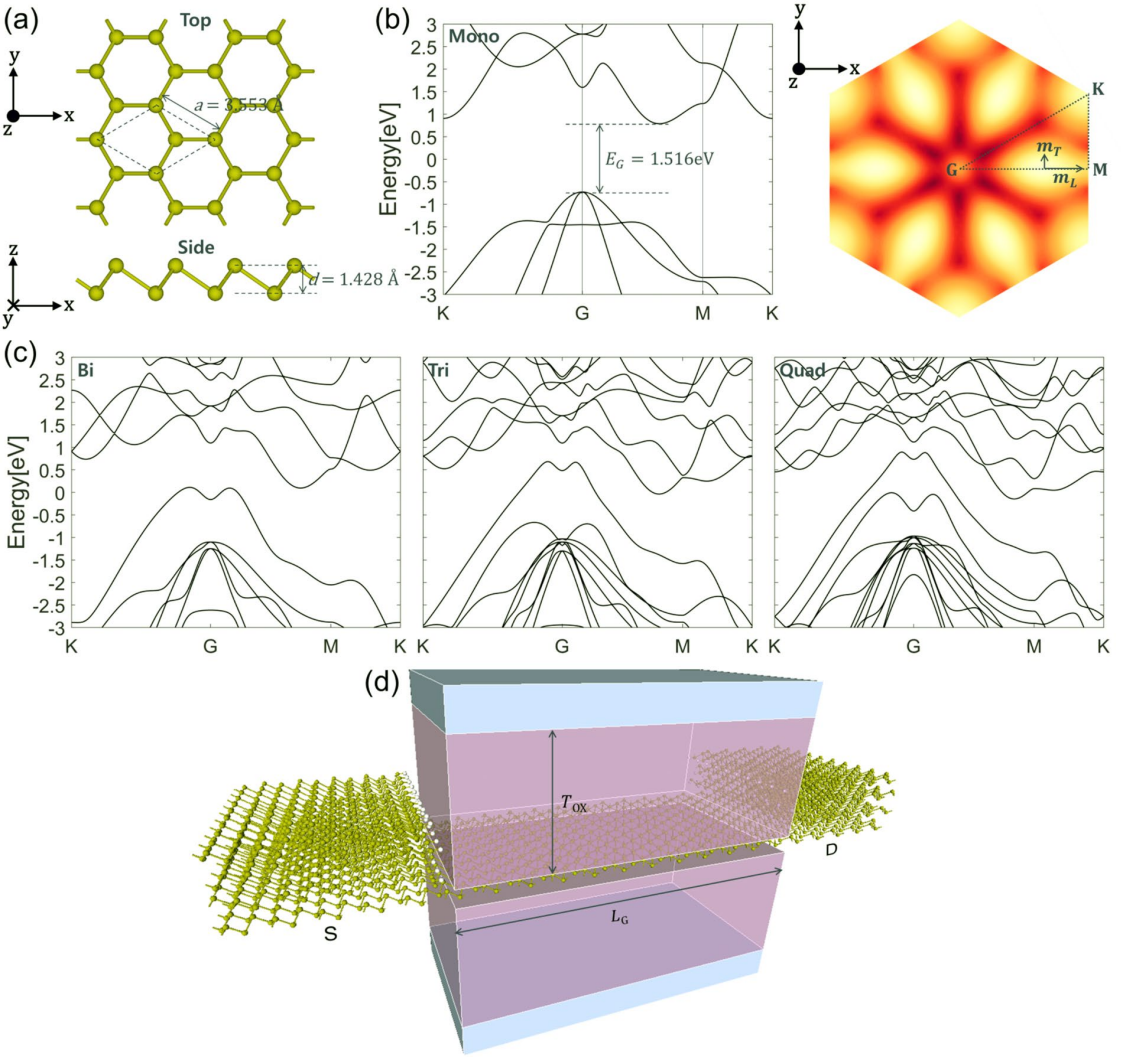
(**a**) Top and side views of monolayer arsenene with primitive unit cell. (**b**) Band structures of monolayer arsenene and energy contour plots of the lowest CB in monolayer arsenene. Effective masses along the longitudinal and transverse directions for one CB valley are indicated. (**c**) Band structures of bilayer, trilayer and quadlayer arsenene along the high symmetric paths in the 1^st^ BZ. The Fermi level is set to zero. (**d**) Schematic view of DG quadlayer/monolayer/quadlayer arsenene heterostructure MOSFETs. Top and bottom HfO_2_ (*κ* = 25) gate oxides thickness *T*_OX_ is 3 nm. Gate length *L*_G_ is ~7 nm. Four multilayers (bilayer, trilayer, quadlayer and hexalayer) are considered to construct multilayer/monolayer/multilayer heterostructures.

We first study band structures of monolayer and multilayer arsenene with the optimized geometric structures. As in Fig. 1(a), monolayer arsenene is in the buckled hexagonal honeycomb lattice with the optimized in-plane lattice constant *a* = 3.553 Å and the buckling height between two sublattices *d* = 1.428 Å in good agreement with the previous studies^15,17,30–33^. In the left of Fig. 1(b), band structures for monolayer arsenene is plotted along the high symmetric points K-Γ-M-K in the hexagonal 1^st^ Brillouin Zone (BZ). Similar to the other DFT calculations^17,30–33^, we observe conduction band minimum (CBM) occurring along the line between Γ and M points and valence band maximum (VBM) at Γ point with the indirect band gap *E*_*G*_ = 1.516 eV. There are several possible choices for exchange correlation potentials and pseudopotentials in the Perdew-Burke-Ernzerhof (PBE) level which yield a somewhat smaller or larger band gap than 1.516 eV as seen in Fig. S1. We choose the combination of exchange correlation potential and pseudopotential resulting in the band gap value 1.516 eV since it is roughly the average. Our band gap value is also close to the previously reported ones (1.48^17^, 1.59^30^, 1.5^31^, 1.6^32^, 1.635^33^ and 1.76^15^ eV) by PBE functionals while smaller compared with the ones (2.0^32^, 2.2^31^ eV) by HSE functionals. Typically, DFT with PBE functionals underestimates the band gap. For several other 2-D materials including MoS_2_, however, band gaps predicted by PBE functionals agree well with the experimental measurements^34^. Moreover, a significant band gap reduction by increasing temperature from 0 K to room temperature in the ambient condition is predicted by the DFT calculation using HSE functionals for monolayer antimonene which shares the similar crystal structure and band structures with monolayer arsenene^24^. Therefore, since we explore device performances at room temperature, our band gap value 1.516 eV is valid for the following device simulations. As shown in the energy contour plot for the lowest CB in the right of Fig. 1(b), we observe six-fold degenerate CB valleys with highly anisotropic effective masses. A longitudinal effective mass from Γ to M and a corresponding transverse effective mass are estimated to *m*_*L*_ = 0.461 × *m*_*e*_ and *m*_*T*_ = 0.174 × *m*_*e*_, respectively. Such anisotropic effective masses usually lead to the strong dependency of device performances on the transport direction. For arsenene, however, we suppose a negligible effect of the transport direction owing to the degenerate CB valleys located between Γ and M points in the hexagonal 1^st^ BZ. As discussed in the anisotropic 2-D material HfS_2_ MOSFETs^35^, aligning one valley in the light effective mass direction is always accompanied by the alignment of the other valleys in the heavy effective mass direction. Therefore, current increase or decrease in one valley is compensated by current decrease or increase in the other valleys, which results in little change in total current. So, we focus on the transport only in the armchair direction. Band structures of multilayers (bilayer, trilayer, quadlayer and hexalayer) are also studied with the optimized geometry. We perform geometry optimizations for bilayer, trilayer, quadlayer and hexalayer, respectively, with the fixed in-plane lattice constant *a* = 3.553 Å obtained from the monolayer calculation. As presented by the band structure of bilayer arsenene in the left of Fig. 1(c), stacking one more layer turns semiconducting monolayer into metallic bilayer by entirely closing the band gap. More bands are induced around the Fermi level in trilayer (in the middle of Fig. 1(c)), quadlayer (in the right of Fig. 1(c)) and hexalayer (not shown) arsenene.

For the device simulation, double-gate (DG) arsenene heterostructure MOSFETs with the intrinsic semiconducting monolayer as channel and the metallic multilayer as source and drain are constructed. We consider four different multilayers (bilayer, trilayer, quadlayer and hexalayer) for the doping-free metallic source and drain which form Schottky junctions at the monolayer/multilayer heterointerfaces. DG quadlayer/monolayer/quadlayer arsenene heterostructure MOSFETs is schematically shown in Fig. 1(d). We choose the HfO_2_ (*κ* = 25) gate oxide thickness *T*_OX_ = 3 nm, the gate length *L*_G_ ≈ 7 nm and the supply voltage *V*_DD_ = 0.7 V which are compatible with the ITRS (International Technology Roadmap for Semiconductors) 2024 requirements for high performance (HP) devices^36^. Periodic boundary conditions are assumed in the device width direction (*y*-direction).

**Figure 2:**
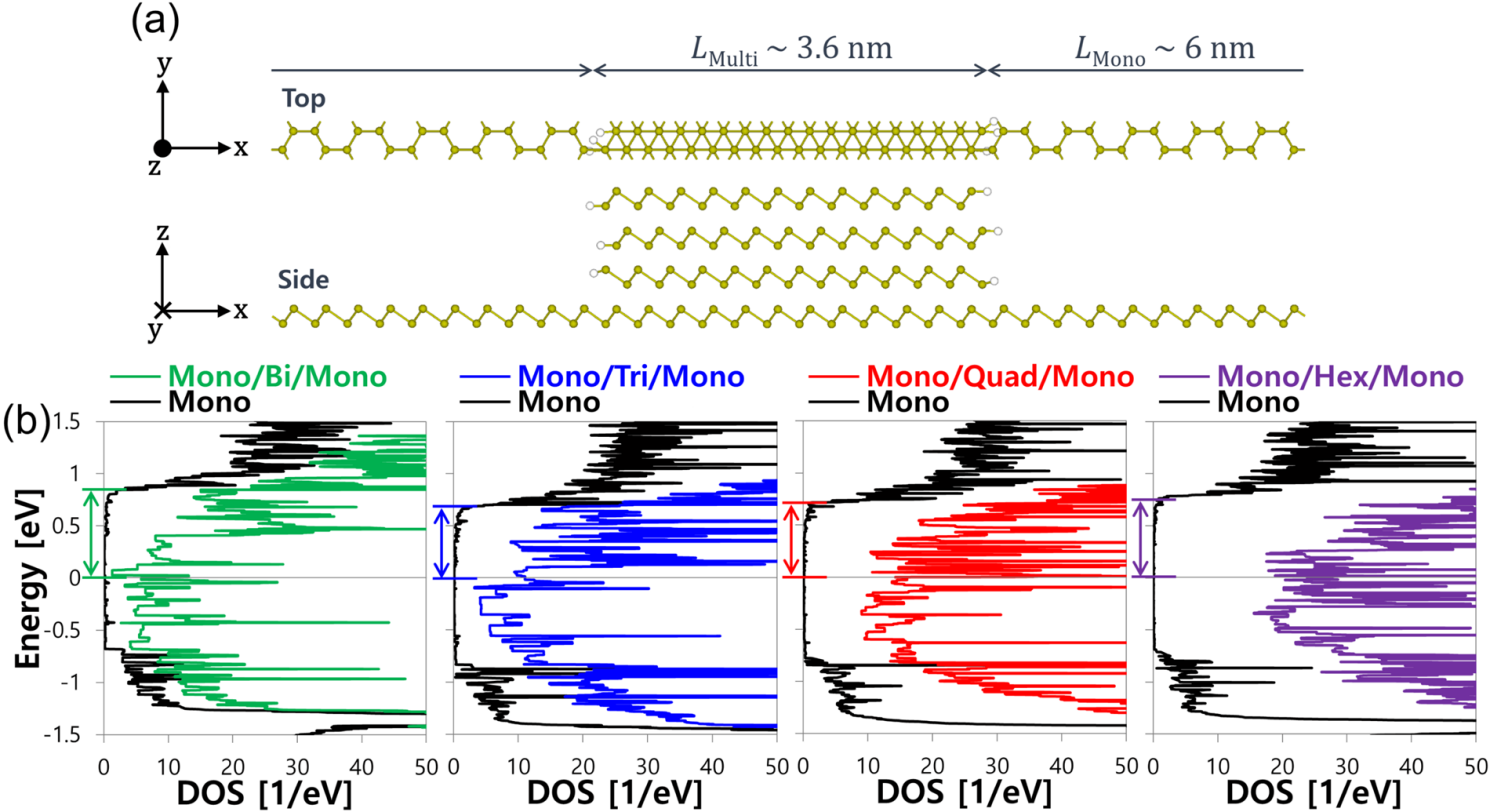
(**a**) Top and side views of monolayer/quadlayer/monolayer heterostructure supercell for DOS calculations. Four monolayer/multilayer/monolayer heterostructures are constructed by combining ~6 nm monolayer with ~4 nm bilayer, trilayer, quadlayer and hexalayer, respectively. (**b**) DOS of monolayer/bilayer/monolayer, monolayer/trilayer/monolayer, monolayer/quadlayer/monolayer and monolayer/hexalayer/monolayer heterostructures. Total DOS and PDOS only from monolayer are plotted together for each heterostructure. Estimated Schottky barrier heights at the monolayer/multilayer heterojunction are indicated by arrows.

We carry out the density of states (DOS) analysis on the semiconducting monolayer/metallic multilayer arsenene heterojunction to understand Schottky barrier (SB) which is critical to the device performance of our proposed MOSFETs. Figure 2(a) shows the supercell for the DOS calculation which is composed of ~6 nm monolayer and ~4 nm multilayer. In the repeated supercell, monolayer/multilayer heterojunctions are formed in the armchair direction (*x*-direction), the transport direction in device simulations, while either monolayer or multilayer is maintained in the zigzag direction (*y*-direction). In the *z*-direction perpendicular to the layer plane, a vacuum region is added on top of the layer to block any interactions between adjacent supercells. At the monolayer/multilayer junction, unsaturated arsenic atoms on the surface of multilayer edges are passivated with hydrogen atoms to prevent the emergence of dangling bond states. Figure 2(b) presents total DOS from the entire supercell for monolayer/bilayer/monolayer (green), monolayer/trilayer/monolayer (blue), monolayer/quadlayer/monolayer (red) and monolayer/hexalayer/monolayer (violet) heterostructures, respectively. To determine the SB height, we plot the projected DOS (PDOS) only from the monolayer in the monolayer/multilayer/monolayer heterostructure together with total DOS in Fig. 2(b). The SB heights *Ф*_SB_ for electrons in multilayer are estimated from the relative positions of CBM with respect to the Fermi level at 0 eV as indicated by arrows in Fig. 2(b). *Ф*_SB_ obtained for the monolayer/bilayer heterojunction is the largest (0.808 eV) among the four heterojunctions. Upon the increase of layer number, *Ф*_SB_ decreases down to 0.682 eV in the monolayer/trilayer and then remains almost same in the monolayer/quadlayer and the monolayer/hexalayer. Total DOS plots represent a monotonic increase of DOS around the Fermi level by the addition of layers, which is consistent with the band structure plots in Fig. 1(d). From the DOS analysis, we may expect more current conduction by adding layers in the source and drain which reduces *Ф*_SB_ and introduces more DOS near the Fermi level and hence increases the tunneling probability and the number of propagating modes at the monolayer/multilayer Schottky junction.

**Figure 3:**
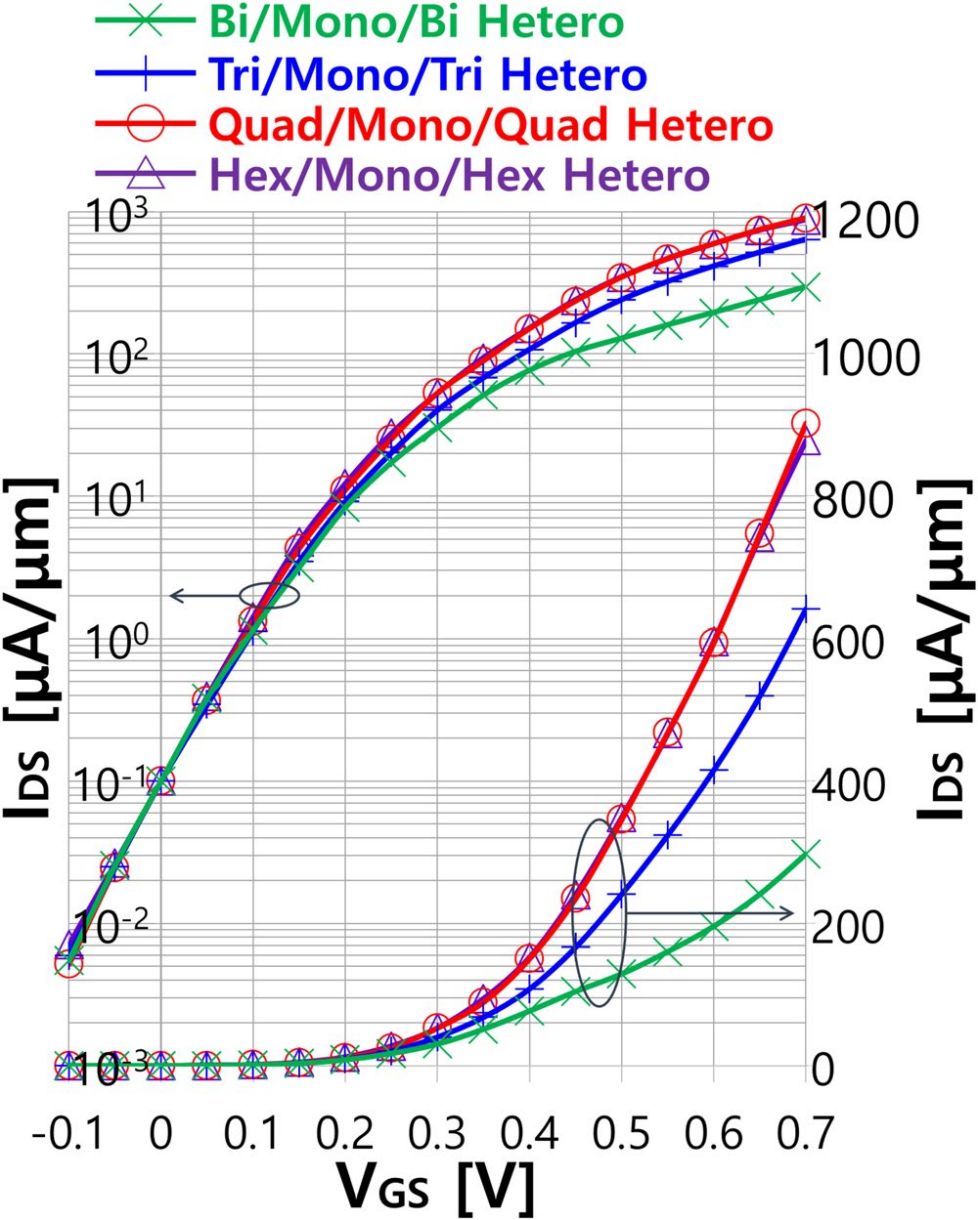
Transfer characteristics of arsenene heterostructure MOSFETs with the bilayer, trilayer, quadlayer and hexalayer source and drain at *V*_DS_ = 0.7 V in logarithm and linear scales.

Transfer characteristics (*I*_DS_-*V*_GS_) of arsenene heterostructure MOSFETs with the bilayer (green), trilayer (blue), quadlayer (red) and hexalayer (violet) source and drain, respectively, at *V*_DS_ = 0.7 V are shown in Fig. 3(a) in logarithmic and linear scales. For the fair comparison, metal gate work functions of each device are adjusted to have the same off-state (*V*_DS_ = *V*_DD_ = 0.7 V and *V*_GS_ = 0 V) current *I*_OFF_ = 0.1 μA/μm according to the ITRS requirements for HP devices^36^. For all devices, irrespective of the number of layers in the source and drain, decent subthreshold slope (*SS*) ≈ 74 mV/dec is observed in ~7 nm gate length devices. As *V*_GS_ increases, transfer characteristics start to exhibit a dependency on the layer number of multilayer. At the on-state (*V*_DS_ = *V*_GS_ = *V*_DD_ = 0.7 V), heterostructure MOSFETs with the bilayer source and drain offers the smallest on-state current *I*_ON_ ≈ 297 μA/μm. With the increase of arsenene layer number, *I*_ON_ continuously improves up to ~642 and ~903 μA/μm with trilayer and quadlayer, respectively, which is consistent with our expectation from the previous DOS analysis. However, further *I*_ON_ enhancement is not achievable by stacking more than four layers in the source and drain. Almost the same level of *I*_ON_ ≈ 882 μA/μm is obtained with the hexalayer source and drain, which suggests an upper limit of *I*_ON_ in our proposed heterostructure MOSFETs at *V*_DD_ = 0.7 V.

**Figure 4:**
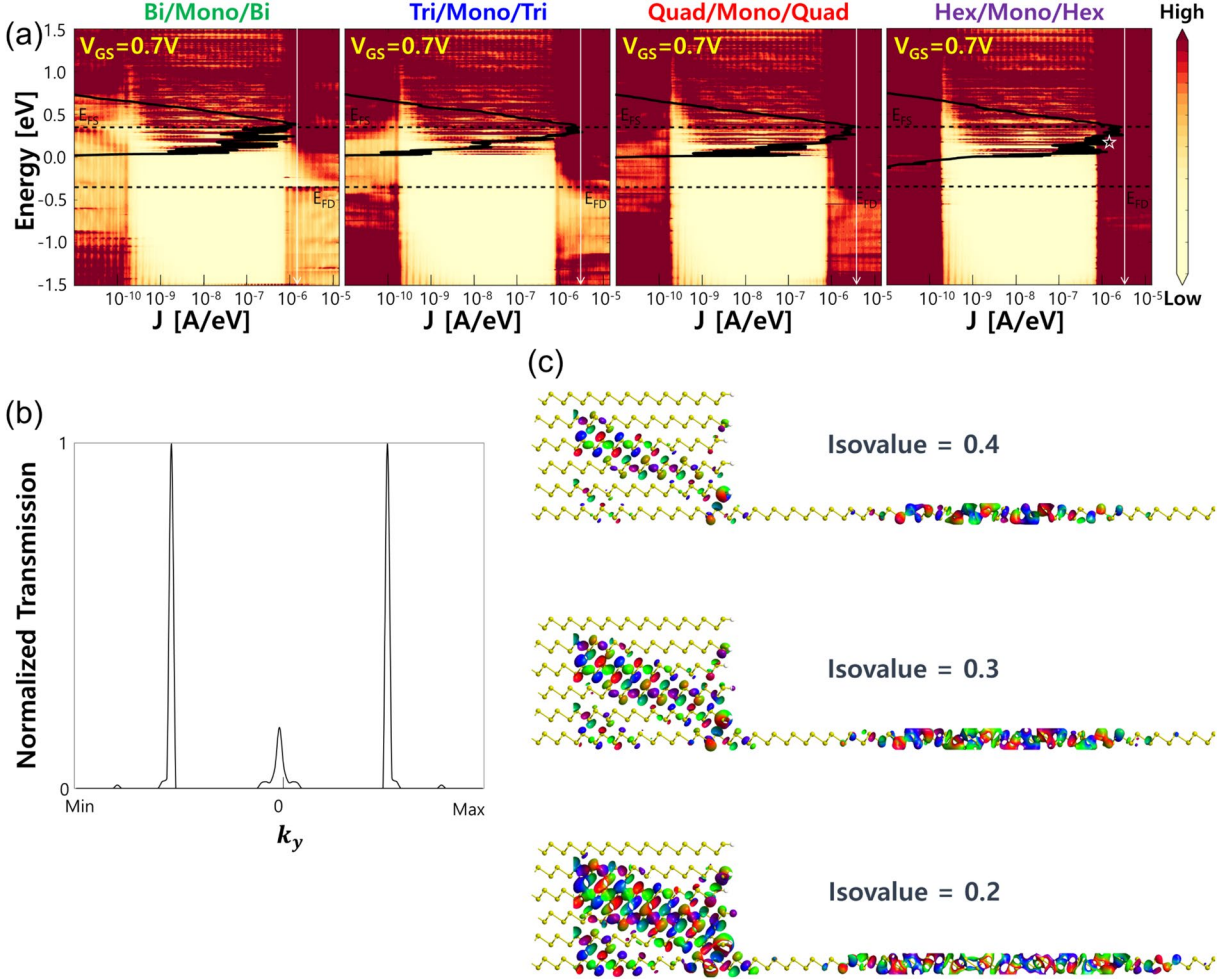
(**a**) LDOS of bilayer/monolayer/bilayer, trilayer/monolayer/trilayer, quadlayer/monolayer/quadlayer and hexalayer/monolayer/hexalayer arsenene heterostructure MOSFETs at *V*_GS_ = 0.7 V with the corresponding energy resolved current densities. (**b**) Transmission spectra in the transverse momentum space *k*_*y*_ at *E* = 0.15 eV for hexalayer/monolayer/hexalayer MOSFETs at *V*_GS_ = 0.7 V (marked with a white star in (**a**)). (**c**) Transmission eigenstates for the transverse momentum *k*_*y*_ yielding the highest transmission in (**b**) at different isovalues of 0.2, 0.3 and 0.4, respectively.

For a deeper understanding of *I*_ON_ dependency on the thickness of multilayer in the source and drain, we show the local DOS (LDOS) at the on-state with the corresponding energy resolved current density (black line) for each heterostructure MOSFETs in Fig. 4(a). *E*_FS_ and *E*_FD_ (black dashed lines) located at 0.35 and −0.35 eV in *y*-axis denote the Fermi levels in the source and drain, respectively. From LDOS plots in Fig. 4(a), we confirm the metallic source and drain and the semiconducting channel with a band gap ~1.516 eV. The energy range in the channel region with bright color represents the absence of states while no such range exists in the source and drain regions which are composed of metallic multilayers. Color of the metallic source and drain regions becomes darker as the layer number increases due to the states originating from the additional layers, which is also predicted from our DOS analysis in Fig. 2(b). At the source/channel junction, the existence of SB is apparent, and the width of SB is narrow due to the large *V*_GS_ = 0.7 V at the on-state, thereby allowing a substantial tunneling current through SB. From bilayer to trilayer, the peak current density indicated by a white arrow significantly boosts up mainly owing to the reduced *Ф*_SB_ as discussed previously with DOS in Fig. 2(b). With quadlayer, even higher peak current density is achieved while no more enhancement with the hexalayer source and drain. To understand this *I*_ON_ saturation upon the increase of layer number in the source and drain, the transmission and the transmission eigenstates at the on-state are explored for heterostructure MOSFETs with the hexalayer source and drain. We focus on the transmission at *E* = 0.15 eV (marked with a white star in Fig. 4(a)) since the primary current conduction is restricted within the upper half of the Fermi window (−0.35 ~0.35 eV). Figure 4(b) shows the transverse momentum *k*_*y*_ resolved transmission, exhibiting the maximum transmission occurring around the half of maximum and minimum values of *k*_*y*_, respectively. The transmission eigenstate corresponding to the maximum transmission in Fig. 4(b) is visualized at different isovalues of 0.2, 0.3 and 0.4, respectively, with the atomistic configuration of the simulated device in Fig. 4(c). At the isovalue of 0.4 (in the top of Fig. 4(c)), the wave function in the source is localized mostly within the third and fourth layers. As approaching the source/channel junction, the wave function gradually moves down to the first and second layers and tunnels through SB and then eventually emerges again near the middle of channel. For the lower isovalues of 0.3 and 0.2 shown in the middle and bottom of Fig. 4(c), respectively, the wave function still resides in the bottom four layers, which suggests the limited contribution of top two layers to the conducting eigenstate. So even if more states become available within the Fermi window with the hexalayer source and drain, only states mostly localized within the bottom four layers could pass through the multilayer source/monolayer channel junction to reach the drain. Therefore, multilayer thicker than quadlayer can conduct almost the same level of current as quadlayer even with the higher DOS in the source and drain.

**Figure 5:**
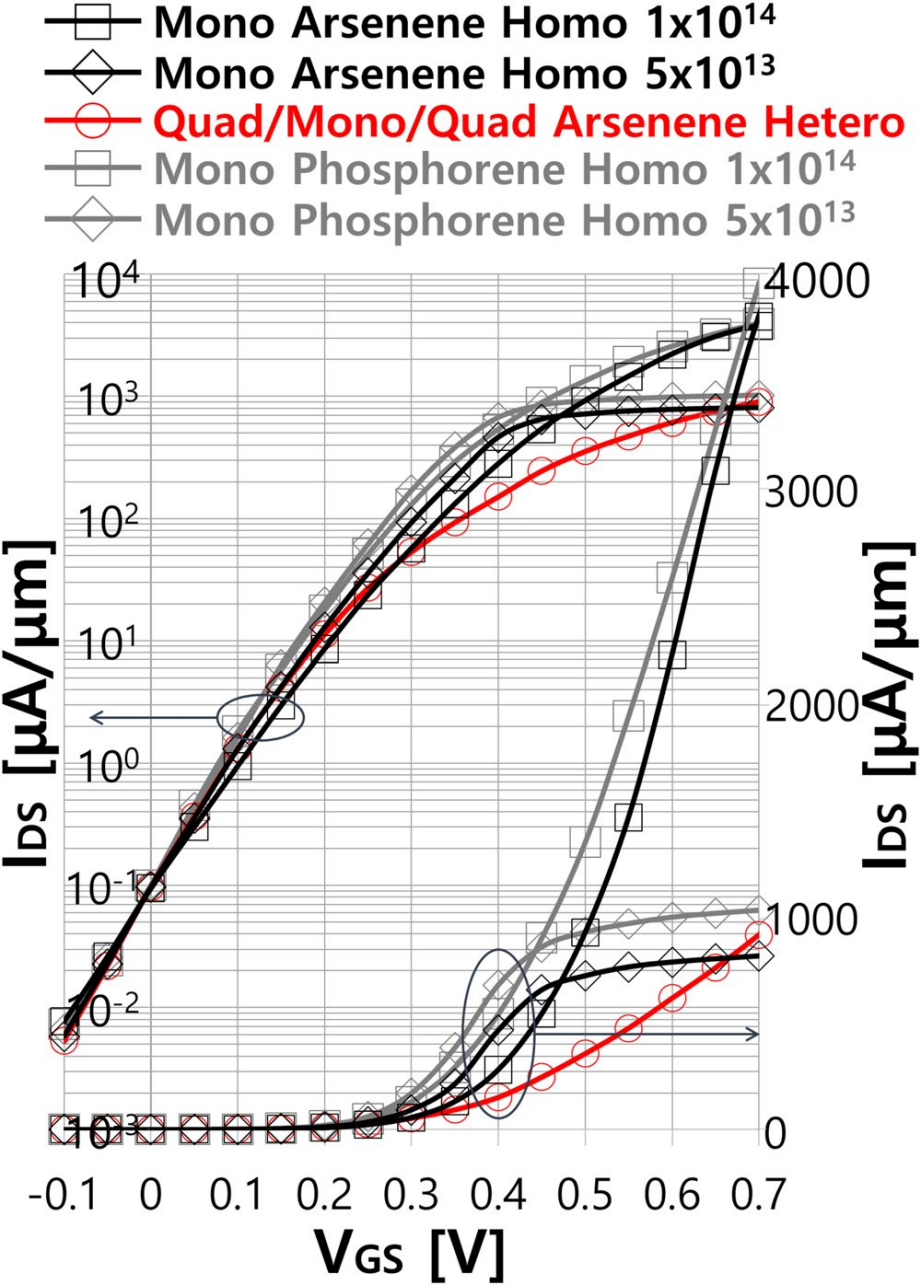
Transfer characteristics of monolayer arsenene and monolayer phosphorene homostructure MOSFETs for the two different doping levels of the source and drain and quadlayer/monolayer/quadlayer arsenene heterostructure MOSFETs.

To evaluate the potential of arsenene heterostructure MOSFETs, it is benchmarked with monolayer arsenene homostructure MOSFETs equipped with the n-type doped source and drain. Two high doping levels of 5 × 10^13^ and 1 × 10^14^/cm^2^ are considered for the source and drain electrodes. Fermi levels corresponding to 5 × 10^13^ and 1 × 10^14^/cm^2^ doping levels are located slightly above and ~100 meV above CB edge, respectively, thereby leading to the degenerately doped monolayer arsenene. For the heterostructure MOSFETs, we focus on the quadlayer source and drain since the best *I*_ON_ is obtained with quadlayer. For the performance assessment against the other 2-D material MOSFETs, we also simulate monolayer phosphorene homostructure MOSFETs with the doping levels of 5 × 10^13^ and 1 × 10^14^/cm^2^. We choose phosphorene since phosphorene has been identified as one of the best candidates for 2-D material MOSFETs among the various 2-D materials due to the highly anisotropic CB and VB valleys^35,37^ as in Fig. S2. Transport calculations for arsenene and phosphorene homostructure MOSFETs with doped reservoirs are performed for the same device geometry (*T*_OX_ = 3 nm and *L*_G_ ≈ 7 nm) within the same simulation setting (GGA PBE functionals, FHI pseudopotential and DZP basis set) as arsenene heterostructure MOSFETs. The armchair direction which exhibits the light effective mass is used for phosphorene homostructure MOSFETs to maximize device performances. Figure 5 compares transfer characteristics of arsenene homostructure MOSFETs for the doping levels of 5 × 10^13^ (black diamond) and 1 × 10^14^/cm^2^ (black square) and arsenene heterostructure MOSFETs (red circle). Transfer characteristics of phosphorene homostructure MOSFETs with 5 × 10^13^ (grey diamond) and 1 × 10^14^/cm^2^ (grey square) doping levels are also shown. In the log scale plot, arsenene homostructure MOSFETs exhibits degraded subthreshold behaviors with *SS* ≈ 80 and 92 mV/dec for the doping levels of 5 × 10^13^ and 1 × 10^14^/cm^2^, respectively, as compared to the heterostructure MOSFETs (*SS* ≈ 74 mV/dec). Even more or similar level of degradation in *SS* (~84 and ~88 mV/dec for 5 × 10^13^ and 1 × 10^14^/cm^2^ respectively) is observed in phosphorene homostructure MOSFETs. On the other hand, linear scale transfer characteristics present significantly better performances in the 1 × 10^14^/cm^2^ doped homostructure MOSFETs in the above threshold region. *I*_ON_ reaches up to ~3800 and ~3950 μA/μm in arsenene and phosphorene homostructure MOSFETs, respectively, more than 4 times higher *I*_ON_ in arsenene heterostructure MOSFETs. For the lower doping level (5 × 10^13^cm^2^), current ramps up more rapidly than in the heterostructure MOSFETs with the increase of *V*_GS_. However, current starts to saturate early around *V*_GS_ ≈ 0.45 V due to the limited supply of electrons from the source while the heterostructure MOSFETs exhibits linearly increasing current. As a result, the heterostructure MOSFETs can eventually provide about 11% larger *I*_ON_ than arsenene and almost comparable *I*_ON_ to phosphorene homostructure MOSFETs, respectively, suggesting that more than a 5 × 10^13^cm^2^ doping is required for arsenene homostructure MOSFETs to outperform arsenene heterostructure MOSFETs. The doping level of 5 × 10^13^cm^2^ corresponds to adding one extra electron for every ~36 As atoms in monolayer arsenene, which is extremely challenging even if the conventional substitutional doping strategy becomes applicable to arsenene.

**Figure 6:**
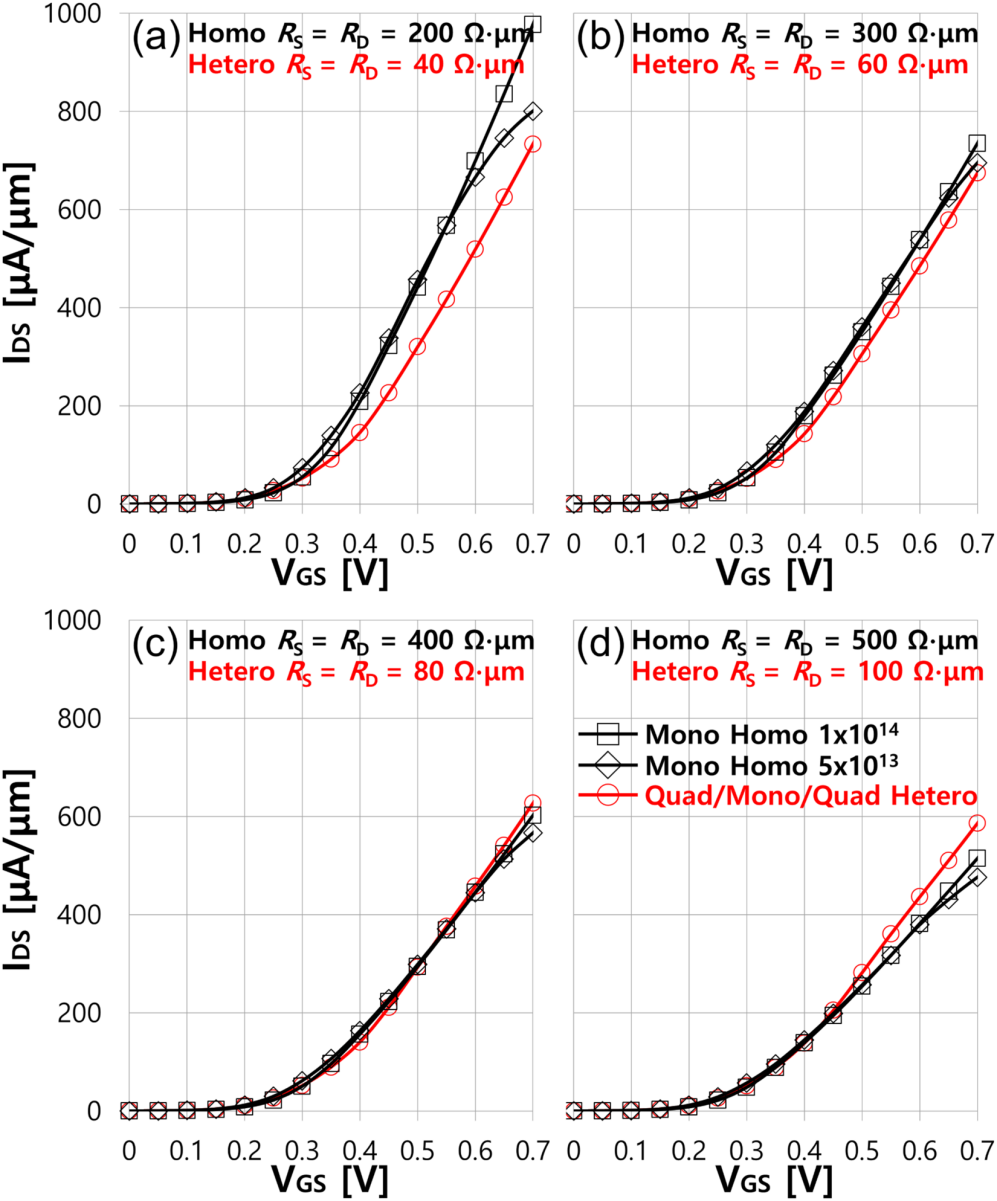
Transfer characteristics of monolayer arsenene homostructure MOSFETs for the two different doping levels of the source and drain and quadlayer/monolayer/quadlayer arsenene heterostructure MOSFETs. *R*_S_ = *R*_D_ = (**a**) 40 and 200 Ω∙μm, (**b**) 60 and 300 Ω∙μm, (**c**) 80 and 400 Ω∙μm and (**d**) 100 and 500 Ω∙μm are included for arsenene heterostructure MOSFETs and monolayer arsenene homostructure MOSFETs, respectively.

In all device simulations discussed so far, the source and drain metal contact regions are excluded. So, our simulation results are valid for the idealized intrinsic devices ignoring any effects from the metal contact. In real devices, metal contacts impose an additional resistance which degrades the device performances. This extrinsic resistance *R*_S/D_ can be characterized by the source and drain resistances *R*_S_ and *R*_D_ (*R*_S/D_ = *R*_S_ + *R*_D_ and *R*_S_ = *R*_D_) connected in series with the source and drain, respectively^38–40^. For more realistic evaluation of the device performances, we add *R*_S/D_ in the postprocessing step to the intrinsic transfer characteristics obtained from the quantum transport simulations. For the postprocessing step, a set of *I*_DS_-*V*_GS_ curves at several different *V*_DS_ is prepared. Then, new *V*_GS,ext_ and *V*_DS,ext_ accounting for the extrinsic metal contact effects are calculated following the equations of *V*_GS,ext_ = *V*_GS,in_ + *I*_DS_*R*_S_ and *V*_DS,ext_ = *V*_DS,in_ + *I*_DS_(*R*_S_ + *R*_D_) where *V*_GS,in_, *V*_DS,in_ and *I*_*D*S_ are the original biases and current, respectively, provided by the quantum transport simulations. Once *V*_GS,ext_ and *V*_DS,ext_ are computed, we collect the *I*_DS_-*V*_GS,ext_ data corresponding to *V*_DS,ext_ = 0.7 V which yields new transfer characteristics at *V*_DS,ext_ = 0.7 V. This method has been adopted in the previous studies providing a good interpretation of experimental data^41–43^. More details of this procedure are well described in ref.^42,43^. We postprocess intrinsic transfer characteristics (Fig. 5) of the homostructure MOSFETs for the different doping levels of 5 × 10^13^and 1 × 10^14^/cm^2^ and the heterostructure MOSFETs with the quadlayer source and drain and then carry out the performance benchmarking again. Different resistance values for *R*_S_ = *R*_D_ = 200, 300, 400 and 500 Ω∙μm are considered for the homostructure MOSFETs. We use the lower resistance values for the heterostructure MOSFETs since it is reasonable to expect a smaller resistance from the metal-to-metal interface than from the metal-to-semiconductor interface. Moreover, a recent experimental study on the device in similar concept with our proposed heterostructure MOSFETs reports dramatic improvement in the contact resistance through the phase engineering^26^ on the source and drain. ref.^26^ demonstrates 2-D material MoS_2_ heterostructure MOSFETs composed of the metallic 1 T phase MoS_2_ for the source and drain electrodes and the semiconducting 2 H phase MoS_2_ for the channel and realizes ~80% reduction in the contact resistance as compared with MoS_2_ homostructure MOSFETs using the semiconducting 2 H phase MoS_2_ for all device regions. Therefore, 20% of the resistance values used in the homostructure MOSFETs (*R*_S_ = *R*_D_ = 40, 60, 80 and 100 Ω∙μm) are adopted in the heterostructure MOSFETs for the fair benchmarking. Postprocessed transfer characteristics with different resistance values *R*_S_ = *R*_D_ = 200/40, 300/60, 400/80 and 500/100 Ω∙μm are plotted respectively in Fig. 6. The source and drain resistances have a negligible effect on the subthreshold behaviors, but strongly influence the on-state current. With *R*_S_ = *R*_D_ = 200 Ω∙μm in Fig. 6(a), *I*_ON_ in the 1 × 10^14^/cm^2^ doped homostructure MOSFETs substantially decreases from its intrinsic *I*_ON_ by more than ~75% while only ~3% degradation with the 5 × 10^13^cm^2^ doping. Such low *I*_ON_ degradation in the 5 × 10^13^cm^2^ doped homostructure MOSFETs is attributed to the early saturation of current observed in Fig. 5. The source resistance reduces the effectively applied *V*_GS_ by *I*_DS_*R*_S_ from the intrinsic *V*_GS_, which results in the significant current decrease for the 1 × 10^14^/cm^2^ doped homostructure MOSFETs exhibiting linearly increasing transfer characteristics as seen in Fig. 5. For the doping level of 5 × 10^13^cm^2^, however, similar level of *I*_ON_ is still maintained as long as the effective *V*_GS_ remains above ~0.45 V due to the current saturation and hence, a limited *I*_ON_ degradation is observed in Fig. 6(a). The heterostructure MOSFETs is relatively less affected by *R*_S/D_ in comparison with the 1 × 10^14^/cm^2^ doped homostructure MOSFETs because the original intrinsic *I*_ON_ is much smaller (~25%), and the reduced *R*_S/D_ is added. However, the homostructure MOSFETs with the 1 × 10^14^/cm^2^ doping can still offer ~33% larger *I*_ON_ than the heterostructure MOSFETs. In Fig. 6(b), *R*_S_ = *R*_D_ = 300 and 60 Ω∙μm for the homostructure and heterostructure MOSFETs, respectively, degrade transfer characteristics further. The degree of degradation is the most severe in the 1 × 10^14^/cm^2^ doped homostructure MOSFETs, which makes *I*_ON_ for all devices within the range of ~10% difference. With *R*_S_ = *R*_D_ = 400 and 80 Ω∙μm in Fig. 6(c), almost same transfer characteristics are predicted for all three devices, but slightly better *I*_ON_ for the heterostructure MOSFETs (~10 and ~4% larger than for the 5 × 10^13^ and 1 × 10^14^/cm^2^ doped MOSFETs, respectively) is expected. The resistance value larger than 400 and 80 Ω∙μm enables the heterostructure MOSFETs to further outperform the homostructure MOSFETs with more than ~15% *I*_ON_ boost as seen in Fig. 6(d).

As discussed previously, there is an uncertainty in the band gap value of monolayer arsenene originating from the different choice of exchange correlation potentials and pseudopotentials in DFT. Such uncertainty could influence device performances of arsenene heterostructure MOSFETs since the SB height *Ф*_SB_ is affected by the band gap size. In general, a smaller band gap (a smaller *Ф*_SB_) improves *I*_ON_, but with the sacrifice of *SS*, while a larger band gap (a larger *Ф*_SB_) degrades *I*_ON_, but leads to the better *SS*. Since our band gap value is roughly the average, our simulations suggest the average performance which we can expect from arsenene heterostructure MOSFETs. Additionally, we note that our ballistic simulations provide an upper limit of device performances. Inclusion of electron-phonon scattering may degrade actual device performances. However, ref.^17^ estimates a few tens of nanometer length for the mean free path limited by the longitudinal acoustic phonon in arsenene. Therefore, in the extremely scaled MOSFETs such as our ~7 nm gate length device, electron-phonon scattering would have a limited impact.

## Conclusions

We present a rigorous analysis for the potential performance of arsenene heterostructure MOSFETs utilizing the exotic property of arsenene, the thickness modulated semiconductor to metal transition. Such an abrupt modification of electronic structures depending on the number of arsenene layers can provide a solution to the fundamental issues in the realization of high performance 2-D material MOSFETs such as the lack of doping technique and the high contact resistance. Exploiting the thickness controlled phase transition in arsenene, the heterostructure MOSFETs could be realized within the same material platform without doping simply by constructing heterostructures consisting of different thicknesses of arsenene layers through the top-down selective etching. Through DFT combined with NEGF simulations, we assess the performance of doping-free arsenene heterostructure MOSFETs and discuss the effect of layer number in the metallic source and drain on the device performances, which suggests the upper limit of intrinsic *I*_ON_ in the heterostructure MOSFETs. Benchmarking with the monolayer arsenene homostructure MOSFETs equipped with the n-type doped source and drain, the heterostructure MOSFETs can intrinsically offer larger *I*_ON_ as well as better *SS* than the homostructure MOSFETs with the 5 × 10^13^cm^2^ doped source and drain in the ~7 nm gate length device. Including the extrinsic metal contact resistance, the heterostructure MOSFETs can possibly surpass even more highly doped homostructure MOSFETs since a lower contact resistance is expected at the interface between contact metal and multilayer metallic arsenene.

## Methods

Electronic band structures, DOS and geometry optimization calculations are carried out with DFT simulation package Atomistix ToolKit (ATK)^38,44,45^. We employ generalized gradient approximation (GGA) and PBE functionals to represent exchange-correlation potentials with double zeta polarized (DZP) basis set^39^. Troullier-Martins type norm-conserving pseudopotential set (FHI [z = 5] DZP) is adopted for arsenene. We include Grimme’s DFT-D2 empirical dispersion correction^40^ to PBE to account for the van der Waals interaction which is known to be important for the accurate description of such layered structures. A *k*-point sampling of 7 × 7 × 1 for the BZ integration is used with a mesh cut-off energy of 45 Hartree. Geometry optimization is performed until the maximum residual force becomes smaller than 0.01 eV/Å.

Device characteristics are investigated through ballistic quantum transport simulations within NEGF formalism using ATK^38,44,45^. Same exchange-correlation functionals, pseudopotentials and basis sets adopted for band structure, DOS and the geometry optimization calculations are also used for the atomistic description of arsenene in device simulations. Since periodic boundary conditions are imposed in the device width direction (*y*-direction), we include different values of transverse momentum *k*_*y*_ in the transport calculation. The electrostatic potential of the device for each bias condition is obtained by solving Poisson’s equation self-consistently with the quantum transport equations. After self-consistent electrostatic potential is determined, total current is calculated by summing the transmitted current over all modes with the Fermi function weight.

## Supplementary information

Doping-Free Arsenene Heterostructure Metal-Oxide-Semiconductor Field Effect Transistors Enabled by Thickness Modulated Semiconductor to Metal Transition in Arsenene
