# Supplementary material for: Doping-Free Arsenene Heterostructure Metal-Oxide-Semiconductor Field Effect Transistors Enabled by Thickness Modulated Semiconductor to Metal Transition in Arsenene
Source: Sci Rep. 2019 Mar 8;9:3988. doi: 10.1038/s41598-019-40675-7 (PMC6408428; doi:10.1038/s41598-019-40675-7)
Supplement: Supplementary file 1 — Doping-Free Arsenene Heterostructure Metal-Oxide-Semiconductor Field Effect Transistors Enabled by Thickness Modulated Semiconductor to Metal Transition in Arsenene [file 41598_2019_40675_MOESM1_ESM.docx]

**Doping-Free Arsenene Heterostructure Metal-Oxide-Semiconductor Field Effect Transistors Enabled by Thickness Modulated Semiconductor to Metal Transition in Arsenene**

Dongwook Seo and Jiwon Chang*

Department of Electrical and Computer Engineering, Ulsan National Institute of Science and Technology (UNIST), Ulsan 44919, South Korea


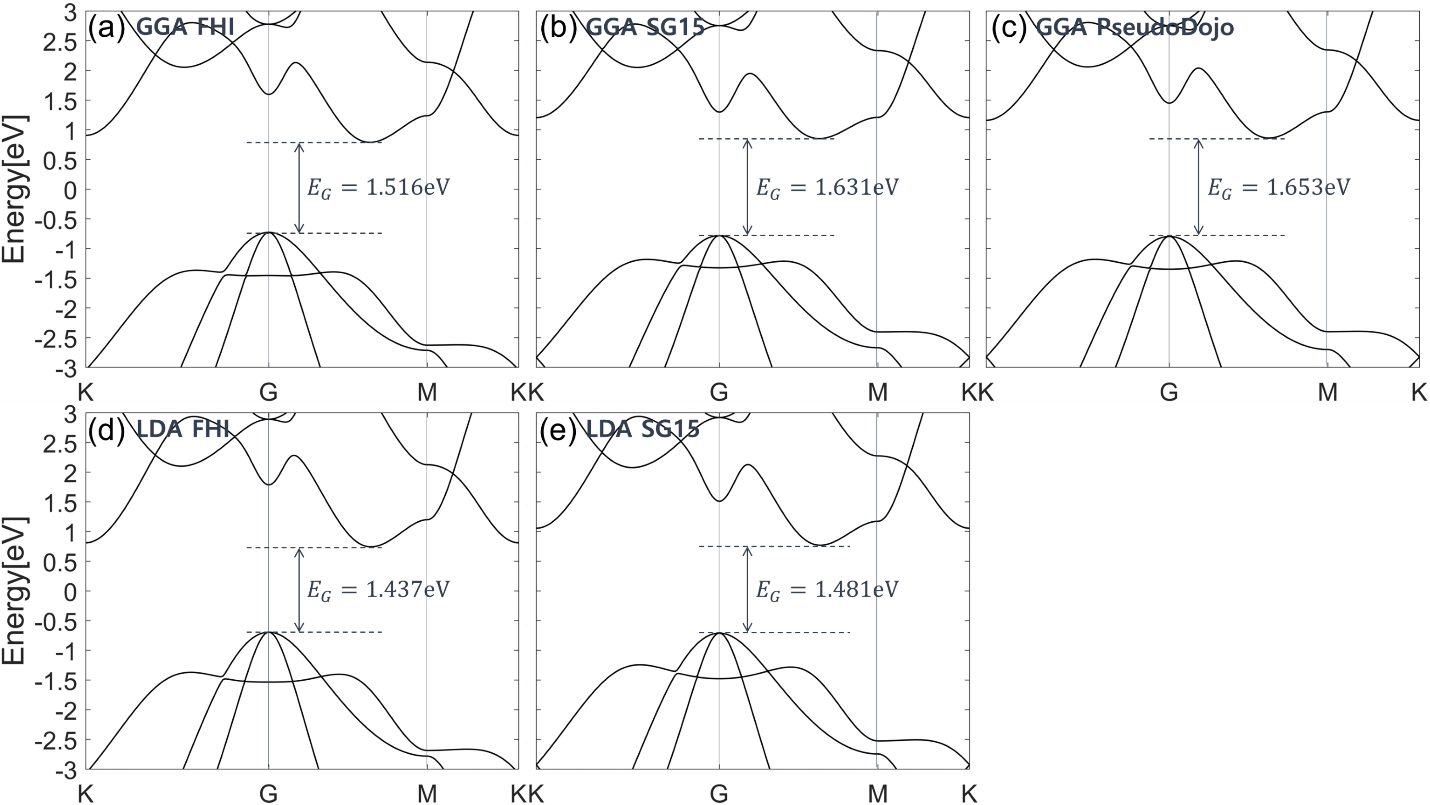


Figure S1. Band structures of monolayer arsenene with different combinations of exchange-correlation potentials and pseudopotentials (a) GGA/FHI, (b) GGA/SG15, (c) GGA/PseudoDojo, (d) LDA/FHI and (e) LDA/SG15.

Band structures of monolayer arsenene are obtained from DFT calculations using various combinations of exchange correlation potentials and pseudopotentials. A *k*-point sampling of 7×7×1 for the BZ integration is used with a mesh cut-off energy of 45 Hartree. Geometry optimization of primitive unit cell is performed with the force convergence criteria less than 0.01 eV/Å.


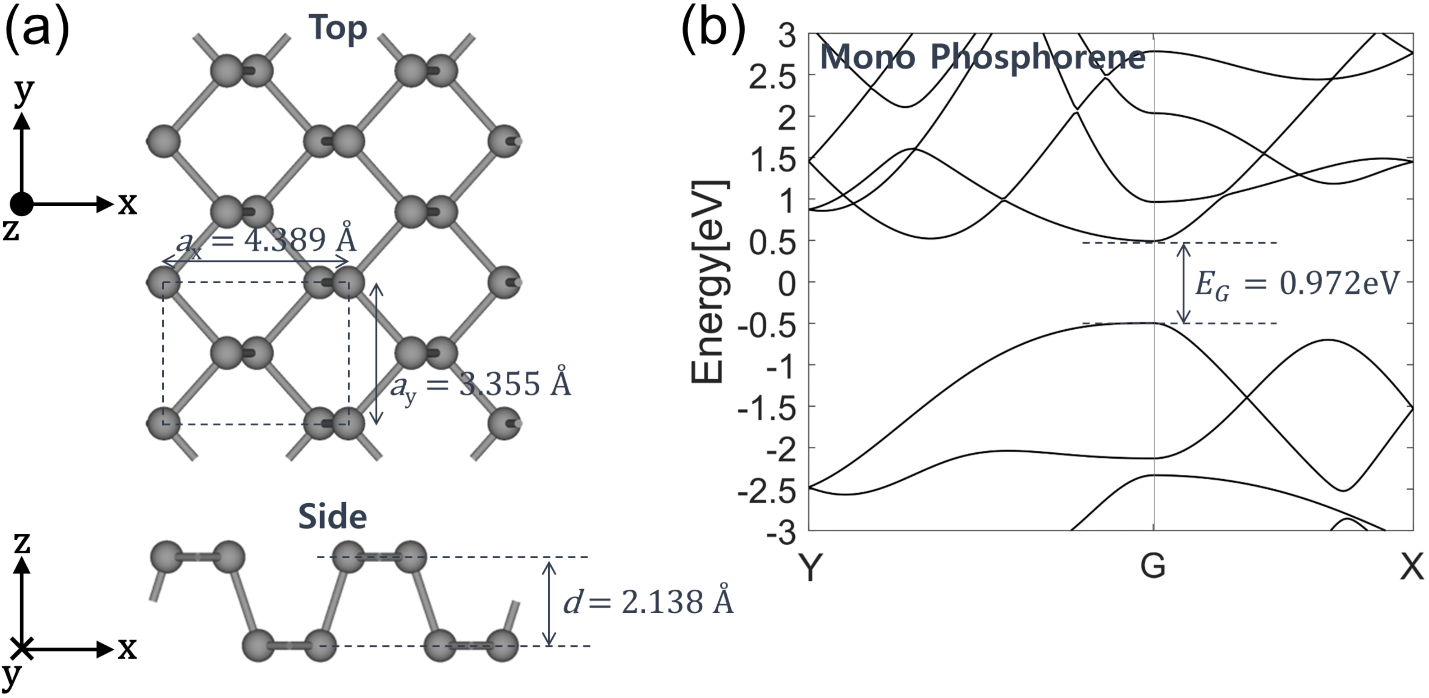


Figure S2. (a) Top and side views of monolayer phosphorene with primitive unit cell. (b) Band structure of monolayer phosphorene along the high symmetric paths in the 1^st^ BZ.

Band structures of monolayer phosphorene are obtained from DFT calculations using GGA PBE functionals, FHI pseudopotential and DZP basis set. Geometry optimization is carried out until the force on each atom are smaller than the threshold 0.01 eV/Å, resulting in the equilibrium lattice constants of 4.389 and 3.355 Å for *a*_x_ and *a*_y_, respectively, and the vertical interatomic distance *d* = 2.138 Å.
